# Effect of restrictive fluid therapy with hydroxyethyl starch during esophagectomy on postoperative outcomes: a retrospective cohort study

**DOI:** 10.1186/s12893-019-0482-z

**Published:** 2019-02-04

**Authors:** Jun-Young Jo, Wook-Jong Kim, Dae-Kee Choi, Hyeong Ryul Kim, Eun-Ho Lee, In-Cheol Choi

**Affiliations:** 1 Department of Anesthesiology and Pain Medicine, Laboratory for Perioperative Outcomes Analysis and Research, Asan Medical Center, University of Ulsan College of Medicine, 88 Olympic-ro 43-gil, Songpa-gu, Seoul, 05505 South Korea; 2 Department of Thoracic and Cardiovascular Surgery, Asan Medical Center, University of Ulsan College of Medicine, Seoul, South Korea

**Keywords:** Esophageal surgery, Intraoperative management, Intravenous fluid, Postoperative outcome

## Abstract

**Background:**

To improve prognosis after esophageal surgery, intraoperative fluid optimization is important. Herein, we hypothesized that hydroxyethyl starch administration during esophagectomy reduce the total amount of fluid infused and it could have a positive effect on postoperative complication occurrence and mortality.

**Methods:**

All consecutive adult patients who underwent elective esophageal surgery for cancer were studied. The primary outcome was the development of composite complications including death, cardio-cerebrovascular complications, respiratory complications, renal complications, gastrointestinal complications, sepsis, empyema or abscess, and multi-organ failure. The relationship between perioperative variables and composite complication was evaluated using multivariable logistic regression.

**Results:**

Of 892 patients analyzed, composite complications developed in 271 (30.4%). The higher hydroxyethyl starch ratio in total fluid had a negative relationship with the total fluid infusion amount (*r* = − 0.256, *P* <  0.001). In multivariable analysis, intraoperatively administered total fluid per weight per hour (odds ratio, 1.248; 95% CI, 1.153–1.351; *P* <  0.001) and HES-to-crystalloid ratio (odds ratio, 2.125; 95% CI, 1.521–2.969; *P* <  0.001) were associated with increased risks of postoperative composite outcomes.

**Conclusions:**

Although hydroxyethyl starch administration reduces the total fluid infusion amount during esophageal surgery for cancer, intravenous hydroxyethyl starch infusion is associated with an increasing risk of postoperative composite complications.

**Electronic supplementary material:**

The online version of this article (10.1186/s12893-019-0482-z) contains supplementary material, which is available to authorized users.

## Background

As surgical techniques and perioperative management have improved, overall mortality from esophagectomy has declined to approximately 10%; however, the morbidity after esophagectomy for esophageal cancer is still approaching to 50% [1–3]. Pulmonary, cardiovascular, and gastrointestinal complications are the most frequent complications [1, 4].

To reduce perioperative morbidity and mortality, various trials have been attempted during the past two decades, such as epidural analgesics, minimal invasive techniques, or intraoperative fluid management based on cutting-edge knowledge [5]. Because different fluids can influence the degree of volume expansion differently and all fluids have their own dose-dependent side effects, appropriate perioperative intravenous fluid selection and volume may be vital for preventing postoperative complications. In case of esophageal surgery in particular, appropriate perioperative fluid management has a significant role for reducing pulmonary complications, which are regarded as the most important cause of death following esophagectomy [5–7]. However, to date, despite extensive studies evaluating the risks and benefits of the types and volume of fluids, the ideal resuscitation fluid or combination of fluids during the perioperative period remains controversial.

Colloids are very effective intravascular volume expanders. Compared with crystalloids, relatively small amounts of a colloid solution can increase plasma volume more during the resuscitation period. However, there are many reports that hydroxyethyl starch (HES) administration results in adverse effects, such as acute kidney injury (AKI) and coagulation disorders [8, 9]. We showed that colloid infusion during esophagectomy is an independent risk factor for postoperative AKI [10]. On the other hand, others have proved that goal directed-fluid therapy with colloids during major abdominal surgery results in better gastrointestinal function, which has benefits on intestinal anastomotic healing [11, 12], and this is contrary to our previous research. Thus, to date there is no consensus about the usefulness or harmfulness of HES infusion to cut down the amount of total administered fluid for overall complications.

In this retrospective analysis, we aimed to verify our hypothesis that HES administration during esophagectomy reduces the total amount of fluid infused and could have a positive result on postoperative complication occurrence and mortality.

## Methods

After approved by the Institutional Review Board of our institution (AMC IRB 2016–1324), we performed a single-center retrospective observational study including all patients aged ≥20 years who underwent elective esophageal surgery at our institution between January 2005 and October 2015. We excluded patients who underwent non-cancer surgery, those who underwent other types of surgery simultaneously, and those with preoperative dialysis or an estimated glomerular filtration rate 60 ml/min/1.73 m^2^. All clinical data about patients was obtained from the Asan Medical Center Esophageal Surgery and Anesthesia Database and from a retrospective review of the computerized patient record system (Asan Medical Center Information System Electronic Medical Record). This study was conducted in accordance with Strengthening the Reporting of Observational Studies in Epidemiology statements [13]. Informed consent was waived by the board.

Esophageal surgery and perioperative management were performed in the standard manner as previously described in details [10]. Briefly, all operations were performed by experienced surgeons and anesthesia was maintained by either volatile anesthetic agent or intravenous anesthetic agent. In all patients, opioid (remifentanil) was administered continuously during surgery and the dosage range was adjusted by assessing hemodynamic parameters. Patients were ventilated to normocapnia (35–45 mmHg) with 50 to 100% oxygen. Conventional parameters including heart rate, continuous arterial pressure, central venous pressure, and urine output were used for hemodynamic and fluid management. To maintain intraoperative intravascular volume, intraoperative fluid replacement was conducted using continuous infusions of crystalloid solution (Hartmann solution; JW Pharmaceutical, Seoul, Korea, or Plasma Solution A; CJ HealthCare Co., Seoul, Korea) at 4 ml/kg/h as maintenance fluid and additional colloid (Voluven® or Volulyte®; Fresenius Kabi, Bad Homburg, Germany) or crystalloid solution based on the patient’s intravascular volume status according to the preference of the attending anesthesiologists. To maintain mean arterial blood pressure between 65 mmHg and 90 mmHg intravenous fluid replacements was performed, firstly. Nevertheless, if the mean blood pressure was kept below 65 mmHg, vasopressor or inotropic agents were administered: bolus injection of phenylephrine (50–100 μg), ephedrine (5–10 mg) or continuous infusion of norepinephrine, dopamine or dobutamine. Transfusion of packed red blood cells was targeted hemoglobin values above 8 g/dl in patients without history of coronary or cerebral artery disease. If with those histories, the targeted hemoglobin level was 10 g/dl. For the pain control, thoracic epidural catheter was inserted before thoracotomy and patient-controlled analgesia with sufentanil started 5 min before the end of surgery. If the insertion of thoracic epidural catheter would not be available due to various reasons, pain control was performed intravenously with fentanyl. After surgery, all patients were transferred to the intensive care unit and discharged to the general ward when their clinical status became stabilized and further intensive monitoring and care were not required.

The primary outcome was composite major complications within 90 days after surgery. The composite 90-day major postoperative complications were defined as the composite outcome of any one or more of the following: 1) death, 2) cardio-cerebrovascular complications, 3) respiratory complications, 4) renal complications, 5) gastrointestinal complications, 6) sepsis, 7) empyema or abscess, or 8) multi-organ failure. A patient experiencing more than one single event was counted only once in the composite outcome. Mortality was defined as death from any cause in-hospital or within 90 days of primary esophageal surgery. Major postoperative complications within 90 days after surgery were defined according to the European Perioperative Clinical Outcome definitions or as previously reported [14, 15]. Cardio-cerebrovascular complications included myocardial infarction, ventricular arrhythmia, application of a mechanical assist device and stroke. Also, respiratory complications were composed of pneumonia for any reason, acute respiratory destress syndrome and respiratory failure requiring mechanical ventilation for more than 48 h. Renal complication was defined according to the Kidney Disease Improving Global Outcomes (KDIGO) criteria and included in the primary outcome if stage 2 or greater. For indirect indicators of perioperative fluid balance status, we used postoperative body weight gain (%). The secondary outcomes were each complication, such as respiratory complication, renal complication, infectious complication, and gastrointestinal complication.

### Statistical analysis

The study sample size was determined to be all patients included in the study and no a priori power analysis was performed. Categorical variables are reported as numbers and percentages and continuous variables are reported as mean ± standard deviation or median with interquartile range. Correlations between total fluid amounts and HES to crystalloid ratio and postoperative weight gain were evaluated using a Pearson’s correlation coefficient. Univariate and multivariable logistic regression analyses were performed to assess the effect of an intraoperative fluid therapy on the postoperative composite outcome. All variables in Table 1 were tested and variables with *P* <  0.20 in univariate analyses were entered into the multivariable analyses. The final model was determined by backward elimination process with a *P* value cutoff of 0.05 as model retention criteria. To evaluate the impact of the fluid variables administered during surgery on the postoperative composite outcome, those variables were separately entered in the final model. In addition, HES-to-crystalloid ratio and the amount of total fluid per weight per hour were entered simultaneously in the final model in order to control the effect of each other and analyze the relationship between each variable and the primary outcome independently. Adjusted odds ratios (ORs) with 95% confidence intervals (CIs) for the multivariable logistic regression were calculated. The discrimination and calibration abilities of each logistic model were assessed by the C statistic and the Hosmer–Lemeshow statistic. All the reported *P* values are 2-sided, and *P* values < 0.05 were considered statistically significant. All data manipulations and statistical analyses were performed using SAS® Version 9.1 (SAS Institute Inc., Cary, NC, USA) software and IBM SPSS Statistics 21.0 (IBM Corp., Armonk, NY, USA).

**Table 1:** Baseline and perioperative characteristics

*N*	Missing	892
*Demographic data*		
Sex (Male/Female)	0	837 (93.8)/55 (6.2)
Age (years)	0	63 [57–69]
Body Mass Index (kg/cm^2^)	0	23.0 ± 2.9
ASA class	0	
I		77 (8.6)
II		795 (89.1)
III		20 (2.2)
*Medical history*		
Diabetes mellitus	0	134 (15.0)
Hypertension	0	321 (36.0)
Current smoker	0	226 (25.3)
Alcohol	0	783 (87.8)
Dyslipidemia	0	71 (8.0)
Ischemic heart disease	0	16 (1.8)
Congestive heart failure	0	29 (3.3)
Cerebrovascular disease	0	32 (3.6)
Peripheral vascular disease	0	23 (2.6)
Chronic obstructive pulmonary disease	0	20 (2.2)
Liver disease	0	95 (10.7)
Atrial fibrillation	0	9 (1.0)
Concurrent Chemo-Radiation Therapy	0	375 (42.0)
*Laboratory data*		
Hematocrit (%)	0	38.3 [34.5–41.4]
Creatinine (mg/dl)	0	0.8 [0.7–0.9]
Estimated GFR^*^ (ml/min/1.73 m^2^)	0	93.8 [85.7–101.1]
Total bilirubin (mg/dl)	0	0.6 [0.4–0.7]
Albumin (g/dl)	0	3.7 [3.5–4.0]
Forced vital capacity (% predicted)	31	93 [84.5–101.0]
Forced expiratory volume in 1 min (% predicted)	31	92 [82.0–101.0]
*Medication*		
ACEI or ARB	0	150 (16.8)
Beta-blocker	0	61 (6.8)
Calcium channel blocker	0	169 (18.9)
Insulin	0	125 (14.0)
Oral hypoglycemic agent	0	89 (10.0)
Statin	0	75 (8.4)
Aspirin	0	51 (5.7)
Plavix	0	15 (1.7)
Diuretics	0	90 (10.1)
*Intraoperative data*		
Operation time (min)	0	339.6 ± 100.5
Crystalloid per weight (ml/kg)	0	29.9 ± 17.4
HES per weight (ml/kg)	0	12.4 ± 8.7
HES-to-crystalloid ratio	0	0.6 ± 0.5
Total fluid per weight per hour (ml/kg/h)†	0	6.1 ± 2.2
Urine output (ml)	0	497.2 ± 417.3
Packed red blood cell (unit)	0	0.3 ± 0.9
None		792 (88.8)
≤ 2 units		74 (8.3)
> 2 units		26 (2.9)
Use of fresh frozen plasma	0	13 (1.5)
Use of platelet concentrate	0	5 (0.6)
*Postoperative data*		
Weight gain (%)	0	0.9 [−0.5–2.3]
Maximal SOFA-c ≥ 2	0	277 (31.1)
Intensive care unit stay (h)	0	24.0 [21.0–45.0]
Hospital stay (days)	0	13.0 [11.0–19.0]

Data are expressed as number of patients (%), mean ± standard deviation, or median [interquartile range]

*: Estimated glomerular filtration rate using Chronic Kidney Disease Epidemiology Collaboration equation

† Total fluid: sum of crystalloid and hydroxyethyl starch during the total anesthetic period

ASA = American Society of Anesthesiology; GFR = glomerular filtration rate; ACEI = angiotensin-converting enzyme inhibitor; ARB = angiotensin receptor blocker; HES = hydroxyethyl starch; SOFA-c: cardiovascular sequential organ failure assessment in the first 24 h

## Results

**Fig. 1:**
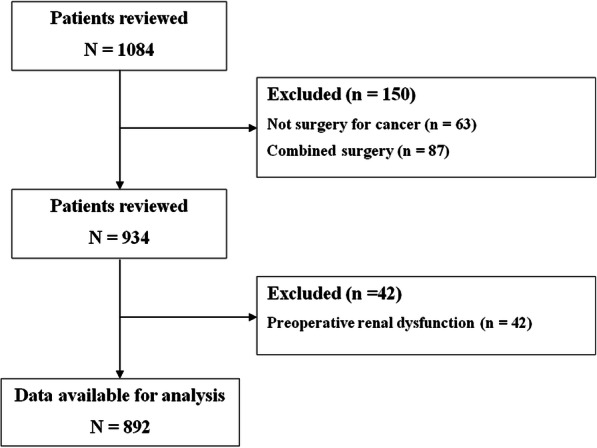
Flow diagram of the study

Of 1084 patients who underwent esophageal surgery for cancer during the study period, 192 met the exclusion criteria, leaving 892 for the analysis (Fig. 1). Composite complications developed in 271 of them (30.4%). The baseline and perioperative characteristics of the study cohort are shown in Table 1. Among the 892 patients, Ivor Lewis operation, McKeown operation, and salvage esophagectomy were performed in 487 (54.6%), 318 (35.7%), and 87 (9.7%) patients, respectively. Totally 183 patients (20.5%) were administered only the crystalloid during surgery. The intraoperative transfusion of packed red blood cells, fresh frozen plasma, or platelet concentrate was performed in 103 patients (11.5%).

Major complications following esophagectomy are shown in Table 2. Among the various complications, renal and respiratory complications comprised the largest portion. Totally, 241 patients (27.0%) suffered AKI based on the KDIGO criteria (stage 1) or greater, and composite respiratory complications, including pneumonia, acute respiratory distress syndrome and respiratory failure occurred in 153 (17.2%). Among those who had pulmonary complication, 71 (46.4%) were accompanied by AKI. In addition, 59 (49.5%) were accompanied by AKI among patients with gastrointestinal complications, and 48 (45.7%) were accompanied by AKI among those with infectious complication. Severe cardiac complications (myocardial infarction, ventricular arrhythmia, and application of a mechanical assist device) occurred in 14 patients.

**Table 2:** Postoperative complications

Complications	Frequency, *n* (%)
Cardio-cerebrovascular	
Myocardial infarction	2 (0.2)
Ventricular arrhythmia	3 (0.3)
Mechanical assist device	9 (1.0)
Stroke	5 (0.6)
Respiratory	
Mechanical ventilation > 48 h	58 (6.5)
Pneumonia	135 (15.1)
Acute lung injury or acute respiratory distress syndrome	26 (2.9)
Renal	
≥ KDIGO stage2	48 (5.4)
Renal replacement therapy	14 (1.6)
Gastrointestinal complications	127 (14.2)
Empyema or abscess	16 (1.8)
Sepsis	97 (10.9)
Multi-organ failure	18 (2.0)
In-hospital death	22 (2.5)
Death within 90 days	34 (3.8)
Composite complications	271 (30.4)

Data are expressed as number of patients (%)

KDIGO = Kidney Disease Improving Global Outcomes

**Fig. 2:**
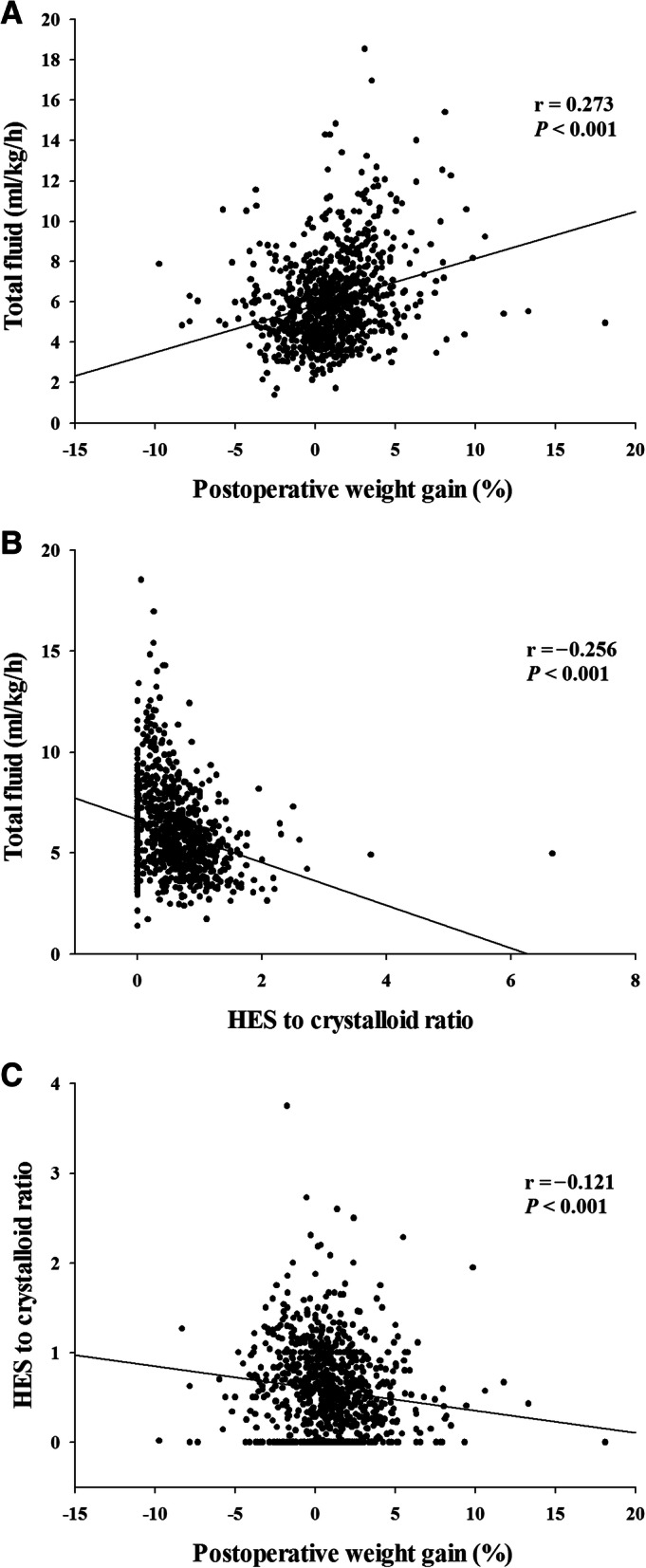
Relationships between total amount of infused fluid and postoperative weight gain (A), total amount of infused fluid and HES-to-crystalloid ratio (B), and HES-to-crystalloid ratio and postoperative weight gain (C). HES = hydroxyethyl starch

The amount of total fluid infused during the operation had a positive relationship with postoperative weight gain (*r* = 0.273, *P* <  0.001, Fig. 2a). The higher ratio of HES in total fluid had a negative relationship with the total amount of fluid infused (*r* = − 0.256, *P* <  0.001, Fig. 2b) and postoperative weight gain (*r* = − 0.121, *P* < 0.001, Fig. 2c). In addition, the predicted probabilities of the incidence of composite complications increased according to the total amount of fluid infused (Additional file 1: Figure S1).

In the univariate analyses, the intraoperatively administered crystalloid per weight (OR, 1.021; 95% CI, 1.004–1.039; *P* = 0.013) and HES per weight (OR, 1.067; 95% CI, 1.041–1.094; *P* < 0.001) were related to an increased risk of postoperative composite outcomes. The multivariable analysis revealed a significantly increased risk of postoperative composite outcomes for the intraoperatively infused HES per weight, HES-to-crystalloid ratio, and total fluid per weight per hour. However, intraoperatively administered crystalloid per weight did not have a significant relationship with postoperative composite outcomes (Table 3). After additional adjustment for the HES-to-crystalloid ratio and total fluid per weight per hour, ORs of the HES-to-crystalloid ratio and total fluid per weight per hour were 2.125 (95% CI, 1.521–2.969; *P* < 0.001) and 1.248 (95% CI, 1.153–1.351; *P* < 0.001), respectively.

**Table 3:** Impact of intravenous fluid administered during esophageal surgery on composite outcomes

	Multivariable Adjusted	
	Odds Ratio (95% CI)	*P* value
Crystalloid per weight (ml/kg)^a^	1.008 (0.998–1.018)	0.101
HES per weight (ml/kg)^a^	1.065 (1.043–1.088)	< 0.001
HES-to-crystalloid ratio^a^	1.595 (1.191–2.136)	0.002
Total fluid per weight per hour (ml/kg/h)^a^	1.178 (1.095–1.268)	< 0.001
HES-to-crystalloid ratio + total fluid^a^		
HES-to-crystalloid ratio	2.125 (1.521–2.969)	< 0.001
Total fluid per weight per hour (ml/kg/h)	1.248 (1.153–1.351)	< 0.001

^a^: Adjusted by ASA class, preoperative hematocrit, preoperative use of diuretics, operation time, and pRBC transfused intraoperatively

CI = confidence interval; HES = hydroxyethyl starch; ASA = American Society of Anesthesiology; pRBC = packed red blood cell

Other risk factors associated with postoperative composite outcomes were preoperative American Society of Anesthesiology class III, preoperatively low hematocrit, intraoperative transfusion of packed red blood cells and longer operation time (Additional file 2: Table S1). Furthermore, an increased HES-to-crystalloid ratio was associated with the increased risk of postoperative respiratory complications, AKI, infectious complications, and gastrointestinal complications (Additional file 3: Table S2).

## Discussion

The key finding of this retrospective observational study on 1084 patients who underwent elective esophageal cancer surgery was that an increased amount of total fluid infused during surgery is associated with an increasing incidence of composite 90-day major postoperative complications. In addition, the high ratio of HES in the total fluid dose is associated with an increase in the incidence of major postoperative complications, despite the benefits of less postoperative weight gain and less requirement of total fluid administration during surgery.

Of the several approaches suggested for lowering postoperative morbidity and mortality, proper intravenous fluid therapy during esophageal surgery is considered to be an integral part of anesthetic management to reduce postoperative surgical and respiratory complications [16]. Several studies have shown that excessive fluid intake during esophageal surgery may be a significant risk factor for the development of postoperative complications, particularly respiratory complications [5, 7, 17]. Therefore, to date, a restrictive fluid regimen that aims achieve a negative perioperative fluid balance is the preferred technique for achieving a good prognosis after undergoing esophagectomy [16]. In our study, we found that increased fluid administration during surgery was associated with an increased risk of postoperative complications; this result supports the current recommendation of fluid restriction in esophageal surgery.

In terms of fluid restriction, an HES solution, which is a colloid solution and generally considered to be a more effective volume expander than crystalloids, could be a good choice for proper intravenous fluid therapy during esophageal surgery [18]. Our study also indicates that the increased use of HES during surgery was associated with lower total intraoperative fluid demand and postoperative weight gain. However, despite its beneficial effect in terms of perioperative negative fluid balance, the increased use of HES was associated with the increased incidence of composite 90-day major postoperative complications. This result is contrary to the results of studies in which a negative fluid balance is considerably associated with a good prognosis after esophagectomy [5, 16, 17, 19]. Thus, a restrictive fluid regimen using HES solution aiming for a negative perioperative fluid balance during esophagectomy could arouse concern regarding some aspects. The HES solution itself could be associated with a poor prognosis, in particular, renal complications. An adverse effect of HES to the kidney is a well-known phenomenon, particularly in case of septic patients in the intensive care unit [20–22]. Some authors argue that HES is not associated with the increased risk of mortality or renal injury in surgical patients without septic conditions, particularly using the latest generation of low molecular weight HES, for example 6% HES 130/0.4 [23]. However, a controversy remains regarding this, and a recent study conducted in patients undergoing cardiac surgery without cardiopulmonary bypass demonstrated that the administration of 6% HES 130/0.4 is also related to the increase in AKI, even though less than 20 mL/kg was used, which is the recommended dose limit [24]. Considering these findings, in our present study, the main cause of perioperative AKI in esophagectomy might be due to the use of HES and not restrictive fluid management itself, and the overall rate of morbidity is likely to be influenced by AKI.

Although it is true that pulmonary complications, which are one of the main causes of death after esophagectomy, constitute most of the relatively severe postoperative complications, the incidence of AKI, including KDIGO stage 1, which is a very mild AKI, is actually higher than that of pulmonary complications. When analyzed with the inclusion of relatively mild AKI, in approximately half of the subjects, AKI was accompanied by other complications, such as respiratory, infectious, and gastrointestinal complications. Even though it was difficult to confirm the order of occurrence and the causal relationship between AKI and other complications, there seemed to be some relevance. According to previous research, the increase in various gastrointestinal complications including gastrointestinal bleeding is related to AKI [25, 26]. In addition, although the incidence of AKI increases in infectious conditions, it has also been shown that AKI itself acts as a risk factor for infection [27]. Taken together, AKI is considered as the main factor among other complications, and the prevention of AKI could be important for a better prognosis after esophagectomy. Therefore, considering such aspects, in esophageal cancer surgery, it would be better to perform restrictive fluid therapy using only crystalloid or using other colloids such as albumin, rather than HES. Additional prospective research on this is necessary.

There were several limitations to our study. First, due to the retrospective nature of our current analyses, our findings should be regarded as a hypothesis generation step, and a causal relationship between the perioperative administration of HES and risk of postoperative complications could not be determined. Although we conducted a multivariable analysis with many variables to obtain reliable results, we cannot exclude some hidden or unmeasured factors that might influence the results. Additionally, we cannot exclude the possibility that the observed relationship between HES administration and postoperative complications may be confounded by indication. In other words, there is a possibility that more HES may have been administered to hemodynamically unstable patients. However, unfortunately, we did not include intraoperative hemodynamic data in our analysis, so we cannot exactly state whether and to what extent confounding by indication affects our results. Thus, our results should be interpreted with caution. Furthermore, we could not consider perioperative analgesic methods such as intravenous or thoracic epidural analgesia. Thoracic epidural analgesia can reduce the incidence of pneumonia and anastomotic leak and the systemic proinflammatory response [28, 29]. Simultaneously, prolonged hypotension due to excess epidural bolus doses is associated with a higher rate of anastomotic leakages [30]. These effects of epidural analgesia could also have influenced the fluid administration strategy. Finally, we could not control the perioperative administration of vasopressors. During esophagectomy, the administered inotropes or vasopressors were chosen according to the anesthesiologists’ preferences. However, each drug might have different impacts on postoperative outcomes. To cope with these limitations, a well-designed randomized controlled study is necessary in the future.

Despite these limitations, this current study is thought to be meaningful because it revealed the relationship between postoperative composite outcomes and specific fluid solutions in esophageal surgery. To our knowledge, there have been many studies on esophageal surgery and fluid therapy, but most have focused on the amount of perioperative fluid regardless of the types of fluid. Our previous study provided results on HES, but the study concerned a limited outcome: postoperative renal dysfunction in esophagectomy [10]. Our current study provides clinicians with an opportunity to reconsider the perioperative administration of HES to reduce the total fluid amount infused during esophagectomy.

## Conclusion

In conclusion, although intravenous HES administration reduces the total amount of fluid infused during esophageal surgery for esophageal cancer, was associated with an increased risk of postoperative composite complications. Therefore, the administration of HES to achieve a negative fluid balance with restrictive fluid management during esophageal surgery may need to be done very carefully.

## Additional files

Additional file 1: **Figure S1.** Predicted probability of composite complications according to the amount of total fluid administered with 95% CI (PDF 31 kb)

Additional file 2: **Table S1.** Multivariable predictors for composite outcomes after esophageal surgery (PDF 113 kb)

Additional file 3: **Table S2.** The odds ratios of HES to crystalloid ratio and total fluid for the various complications (PDF 125 kb)

## Abbreviations

AKI: Acute kidney injury
CI: Confidence interval
HES: Hydroxyethyl starch
KDIGO: Kidney Disease Improving Global Outcomes
OR: Odds ratio
