# Supplementary material for: Effect of restrictive fluid therapy with hydroxyethyl starch during esophagectomy on postoperative outcomes: a retrospective cohort study
Source: BMC Surg. 2019 Feb 4;19:15. doi: 10.1186/s12893-019-0482-z (PMC6360773; doi:10.1186/s12893-019-0482-z)

Figure S1. Predicted probability of composite complications according to the amount of total fluid administered with 95% CI

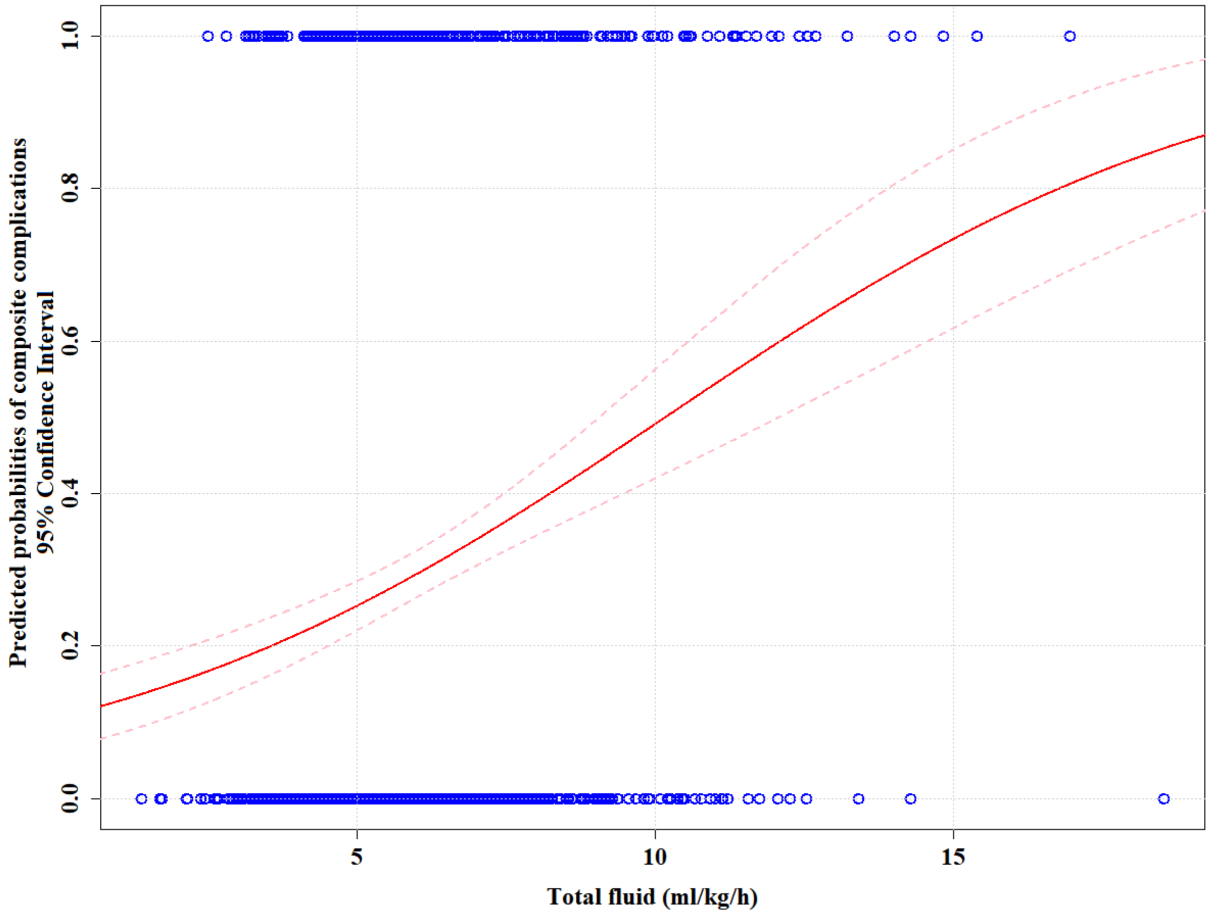

Supplement: Supplementary file 1 — Figure S1. Predicted probability of composite complications according to the amount of total fluid administered with 95% CI (PDF 31 kb) [file 12893_2019_482_MOESM1_ESM.pdf]
