# Supplementary material for: Effect of restrictive fluid therapy with hydroxyethyl starch during esophagectomy on postoperative outcomes: a retrospective cohort study
Source: BMC Surg. 2019 Feb 4;19:15. doi: 10.1186/s12893-019-0482-z (PMC6360773; doi:10.1186/s12893-019-0482-z)
Supplement: Supplementary file 2 — Table S1. Multivariable predictors for composite outcomes after esophageal surgery (PDF 113 kb) [file 12893_2019_482_MOESM2_ESM.pdf]

Table S1. Multivariable predictors for composite outcomes after esophageal surgery

**Predictor**	**Odds Ratio (95% CI)**	***P* value**
ASA class II	1.801 (0.970–3.344)	0.063
ASA class III	4.455 (1.392–14.259)	0.012
Preoperative hematocrit (%)	0.941 (0.909–0.975)	0.001
Preoperative use of diuretics	0.583 (0.327–1.037)	0.067
Operation time (minutes)	1.003 (1.002–1.005)	< 0.001
Transfusion of packed red blood cells of ≤ 2 units	1.735 (1.001–3.009)	0.050
Transfusion of packed red blood cells of > 2 units	3.019 (1.113–8.191)	0.030
Hydroxyethyl starch to crystalloid ratio	2.125 (1.521–2.969)	< 0.001
Total fluid per weight per hour (mL/kg/h)	1.248 (1.153–1.351)	< 0.001

Hosmer-Lemeshow test; P = 0.487, C statistic = 0.730.

CI = confidence interval; ASA = American Society of Anesthesiology.
