# Supplementary material for: Effect of restrictive fluid therapy with hydroxyethyl starch during esophagectomy on postoperative outcomes: a retrospective cohort study
Source: BMC Surg. 2019 Feb 4;19:15. doi: 10.1186/s12893-019-0482-z (PMC6360773; doi:10.1186/s12893-019-0482-z)
Supplement: Supplementary file 3 — Table S2. The odds ratios of HES to crystalloid ratio and total fluid for the various complications (PDF 125 kb) [file 12893_2019_482_MOESM3_ESM.pdf]

**Table S2**. The odds ratios of HES to crystalloid ratio and total fluid for the various complications

| | Multivariable Adjusted | |
|---|---|---|
| | Odds Ratio (95% CI) | *P*-value |
| Composite respiratory complication* | | |
| HES to crystalloid ratio | 1.517 (1.081–2.130) | 0.016 |
| Total fluid per weight per hour (ml/kg/h) | 1.208 (1.108–1.316) | < 0.001 |
| Acute kidney injury based on the KDIGO criteria† | | |
| HES-to-crystalloid ratio | 1.776 (1.243–2.539) | 0.002 |
| Total fluid per weight per hour (ml/kg/h) | 1.002 (0.908–1.105) | 0.973 |
| Composite infectious complication‡ | | |
| HES-to-crystalloid ratio | 1.973 (1.308–2.977) | 0.001 |
| Total fluid per weight per hour (ml/kg/h) | 1.123 (1.011–1.247) | 0.030 |
| Gastrointestinal complication** | | |
| HES to crystalloid ratio | 3.046 (1.913–4.850) | < 0.001 |
| Total fluid per weight per hour (ml/kg/h) | 1.261 (1.144–1.390) | < 0.001 |

*: adjusted by history of COPD and liver disease, preoperative serum albumin levels, preoperative FVC (%), operation time, and pRBC transfused intraoperatively.

†: adjusted by body mass index, history of diabetes mellitus, preoperative estimated glomerular filtration rate, preoperative serum total bilirubin levels, preoperative ejection fraction, and operation time.

‡: adjusted by history of peripheral vascular disease and COPD, preoperative use of statin, preoperative FVC (%), operation time, and pRBC transfused intraoperatively.

**: adjusted by history of dyslipidemia, preoperative hematocrit, preoperative serum total bilirubin levels, preoperative use of diuretics, operation time, and pRBC transfused intraoperatively.

CI = confidence interval; HES = hydroxyethyl starch; KDIGO = Kidney Disease: Improving Global Outcomes classification; COPD = chronic obstructive pulmonary disease; FVC (%) = forced vital

capacity (% predicted); pRBC = packed red blood cell.
